# Primary Immunodeficiency in Children With Autoimmune Cytopenias: Retrospective 154-Patient Cohort

**DOI:** 10.3389/fimmu.2021.649182

**Published:** 2021-04-22

**Authors:** Emma Westermann-Clark, Cristina Adelia Meehan, Anna K. Meyer, Joseph F. Dasso, Devendra Amre, Maryssa Ellison, Bhumika Patel, Marisol Betensky, Charles Isaac Hauk, Jennifer Mayer, Jonathan Metts, Jennifer W. Leiding, Panida Sriaroon, Ambuj Kumar, Irmel Ayala, Jolan E. Walter

**Affiliations:** ^1^ Division of Allergy and Immunology, Department of Pediatrics, Morsani College of Medicine, University of South Florida, Tampa, FL, United States; ^2^ Division of Allergy and Immunology, Department of Medicine, Morsani College of Medicine, University of South Florida, Tampa, FL, United States; ^3^ Division of Allergy and Immunology, Department of Pediatrics, National Jewish Health, Denver, CO, United States; ^4^ Graduate Medical Education, University of Colorado, Denver, CO, United States; ^5^ Department of Biology, University of Tampa, Tampa, FL, United States; ^6^ Cancer and Blood Disorders Institute, Johns Hopkins All Children’s Hospital, St Petersburg, FL, United States; ^7^ Division of Hematology, Department of Pediatrics Johns Hopkins All Children’s Hospital, St. Petersburg, FL, United States; ^8^ Division of Allergy/Immunology, Department of Pediatrics Johns Hopkins All Children’s Hospital, St. Petersburg, FL, United States; ^9^ Research Methodology and Biostatistics Core, Morssani College of Medicine, University of South Florida, Tampa, FL, United States; ^10^ Division of Allergy and Immunology, Massachusetts General Hospital for Children, Boston, MA, United States

**Keywords:** autoimmune cytopenia, primary immunodeficiency, Evans syndrome, immune dysregulation, anemia, thrombocytopenia, neutropenia

## Abstract

**Background:**

Primary immunodeficiency is common among patients with autoimmune cytopenia.

**Objective:**

The purpose of this study is to retrospectively identify key clinical features and biomarkers of primary immunodeficiency (PID) in pediatric patients with autoimmune cytopenias (AIC) so as to facilitate early diagnosis and targeted therapy.

**Methods:**

Electronic medical records at a pediatric tertiary care center were reviewed. We selected 154 patients with both AIC and PID (n=17), or AIC alone (n=137) for inclusion in two cohorts. Immunoglobulin levels, vaccine titers, lymphocyte subsets (T, B and NK cells), autoantibodies, clinical characteristics, and response to treatment were recorded.

**Results:**

Clinical features associated with AIC-PID included splenomegaly, short stature, and recurrent or chronic infections. PID patients were more likely to have autoimmune hemolytic anemia (AIHA) or Evans syndrome than AIC-only patients. The AIC-PID group was also distinguished by low T cells (CD3 and CD8), low immunoglobulins (IgG and IgA), and higher prevalence of autoantibodies to red blood cells, platelets or neutrophils. AIC diagnosis preceded PID diagnosis by 3 years on average, except among those with partial DiGeorge syndrome. AIC-PID patients were more likely to fail first-line treatment.

**Conclusions:**

AIC patients, especially those with Evans syndrome or AIHA, should be evaluated for PID. Lymphocyte subsets and immune globulins serve as a rapid screen for underlying PID. Early detection of patients with comorbid PID and AIC may improve treatment outcomes. Prospective studies are needed to confirm the diagnostic clues identified and to guide targeted therapy.

## Introduction

Autoimmune cytopenias (AICs), including autoimmune hemolytic anemia (AIHA), immune thrombocytopenia (ITP), autoimmune neutropenia (AIN), and their combinations (Evans syndrome [ES]), result from immune dysregulation targeting self-antigens on blood cells (1). AICs are common immunological presentations among pediatric patients (2, 3) and most cases self-resolve or respond to first-line therapy such as corticosteroids or intravenous immunoglobulins (IVIG) (4, 5). In some cases, AICs may indicate serious underlying immune dysregulation preceding the presentation of primary immunodeficiency disorders (PIDs) (3, 6–9). There is increasing awareness that AIC may be a presenting symptom of PID, particularly among patients with ES (10). Studies in patients with ES reveal a variety of underlying PIDs including combined B and T cell abnormalities (combined immunodeficiency [CID]) and T regulatory cell (Treg) defects (3, 10–12). Treatment-refractoriness is another hallmark of autoimmune cytopenia with underlying PID (4, 9, 13, 14).

First line therapy for AIHA and ITP usually includes corticosteroids and/or high dose IVIG, as mentioned above. Patients with underlying PID often require second and third line therapy, and sometimes are refractory to all treatment (8). The care of these patients could be improved by targeted therapy. Targeted therapy can be prescribed only after attaining a diagnosis of underlying PID and understanding the disease mechanism (3, 9, 15–19).

In this retrospective study, we compared patients with AIC alone to patients with both AIC and PID. We examined the time to diagnosis of PID in the setting of AIC, clinical and laboratory features associated with underlying PIDs, and responses to treatment. We identified clinical signs and immunological markers that could enable early detection of PID among patients who present initially with AIC. This manuscript serves as a foundation for a forthcoming prospective AIC study at our center.

## Methods

**Figure 1:**
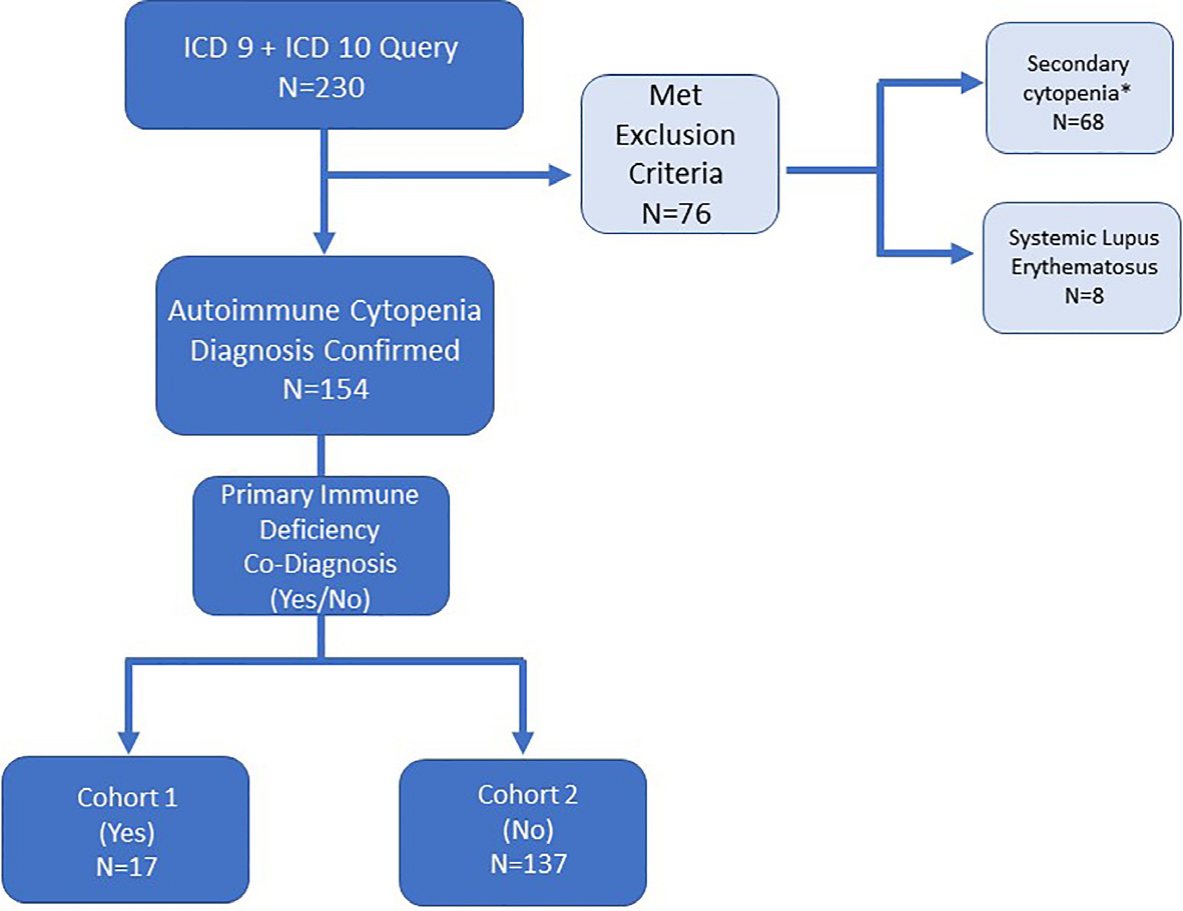
Electronic medical record search strategy and patient inclusion/exclusion criteria for patients with autoimmune cytopenia. ICD-9 and ICD-10 diagnosis codes queried refractory cytopenia with multi-lineage dysplasia (D46.A), autoimmune hemolytic anemia (D59.1, 283.0), acquired hemolytic anemia unspecified (D59.9, 283.9), immune thrombocytopenic purpura (D69.3, 287.31), autoimmune neutropenia (D70.8, 288.09), Evans syndrome (D69.41, 287, 32), and disease of blood and blood-forming organs unspecified (D75.9). *Secondary cytopenias defined as cytopenia caused by bone marrow or solid organ transplantation, malignancy or medication-induced.

This single institution retrospective study was approved by the Johns Hopkins All Children’s Hospital Institutional Review Board (IRB00103900). Data were collected from clinic visits and/or hospital admissions from July 1, 2013 to June 30, 2016. Patients were identified by International Classification of Disease (ICD) codes. An initial electronic medical record query used ICD-9 and ICD-10 codes for autoimmune cytopenias was performed as outlined in **Figure 1**. A second query searched for patients with diagnoses of autoimmune lymphoproliferative syndrome (ALPS) or other lymphoproliferative syndrome but did not yield any unique additional medical record numbers (MRNs). Secondary cytopenias including bone marrow or solid organ transplant, malignancy, and medication-induced cytopenias were excluded upon chart review (**Figure 1**). Immune dysregulation resulting in autoimmunity such as AIC can occur on a background of many primary hematologic disorders, so these were not necessarily excluded (i.e. bone marrow failure syndromes). Cases required detailed chart review and were excluded if an AIC was not present in addition to the primary hematologic disorder. Systemic lupus erythematosus (SLE) patients were excluded because cytopenias are part of the diagnostic criteria for SLE, and we did not want to artificially enrich the dataset with SLE patients by searching specifically for SLE diagnostic codes. However, in light of new knowledge regarding monogenic SLE and overlap with PID, we might have opted to include these patients if we were beginning the study today. Overlap between PID and SLE is addressed further in the discussion section.

For each unique MRN identified, patients were grouped into two cohorts after detailed chart review: (1) co-diagnoses of AIC and PID, or (2) single diagnosis of AIC. AIC diagnosis was based on laboratory data and clinical notes reviewed by two clinical investigators, and a subject was included only when both investigators agreed. In some cases, it was necessary to contact the treating physician to confirm diagnosis of AIC.

For patients in the AIC-PID group, PID was confirmed by laboratory tests for immune phenotype, review of clinician notes and genetic testing, if available. Data collected included patient demographics, medical history, clinical symptoms, laboratory results, and medications. Data regarding the use and outcome of first- and second-line therapies were also collected.

PID encompasses patients with CVID (common variable immune deficiency) or CID. CVID was defined as hypogammaglobulinemia (low IgG with or without low IgA and/or IgM adjusted for age) and poor response to vaccine antigens. CID was defined as evidence of B-cell abnormality (hypogammaglobulinemia, CVID, or poor response to polysaccharide antigens) plus evidence of T-cell abnormality (T-cell lymphopenia and/or reduced lymphocyte proliferation to mitogens or antigens).

Patients were included in the PID group if they had abnormal immune phenotyping and/or genetic testing consistent with PID. Because this is a retrospective study (chart review), objective measures (laboratory values) were deemed the most appropriate way to identify patients with PID. Next, on review of clinician notes, suspicion for PID was confirmed or rejected. For example, an isolated low lymphocyte count in the setting of corticosteroid treatment would not be classified as PID without additional evidence to support PID. Clinical laboratory results were interpreted using reported normal laboratory-reported ranges, except for lymphocyte subsets and immunoglobulin levels, which were compared to age-appropriate normal values (20, 21).

Immunological phenotyping data were collected for all patients for whom the laboratory tests had been performed on a clinical basis. It should be noted that patients who were followed by non-immunologists often did not have complete immune phenotyping. Immune phenotyping includes T, B and natural killer (NK) cell enumeration based on flow cytometry markers of CD3/CD4/CD8 (T cell subsets), CD56 (NK cells), and CD19 (B cells), immunoglobulin levels (IgG, IgA, and IgM), and vaccine titers (recall antigens of *Streptococcus pneumoniae*, diphtheria, and tetanus). Cellular immune function was tested by lymphocyte proliferation to mitogens (phytohemagglutinin, concanavalin and pokeweed) and antigens (candida and tetanus). Autoantibodies to red blood cells (Coombs test), platelets and neutrophils were also collected when available.

Therapeutic response was scored for patients who received treatment using the following criteria: ‘no’ = no clinical response to the intervention or side effects were limiting; ‘partial’ = some clinical improvement but therapeutic escalation was required for stabilization; or ‘full’ = clinical improvement and no subsequent escalation required for stabilization. Response to therapy was evaluated by two authors; when clarification was necessary, the treating physician was contacted. First line therapies included corticosteroids, IVIG and Rho(D) immune globulin (Winrho^®^ [Saul Therapeutics, Roswell, GA, US] in the case of ITP). Rituximab was considered second line. Third line therapies included mycophenolate mofetil (MMF) and cyclosporine. Thrombopoietin receptor agonists (eltrombopag and romiplostim) were considered adjunct therapies. Fourth line therapy was splenectomy. Patients who did not respond to first-line therapy were deemed “refractory cases.” Because ITP is relatively common and can be transient in nature, we specifically examined treatment refractoriness of ITP cases in each group.

## Statistical Analysis

Demographic and patient characteristics were summarized as mean and standard deviation (SD) for continuous variables and as proportions for categorical variables. The difference across compared groups for categorical variables was assessed using Pearson Chi-Square test, and for continuous variables, independent samples t-test was used. Adjusted associations were assessed using binary logistic regression. The statistical significance for all comparisons was set at 5%. All data analysis was performed using Statistical Package for Social Sciences (SPSS) version 25/26.

## Results

### Electronic Medical Record Search

The initial search for patients with a diagnosis of AIC based on ICD-9 or ICD-10 codes yielded 230 MRNs (**Figure 1**). After chart review, patients with secondary cytopenias linked to bone marrow or solid organ transplantation, malignancy, or medication-induced process were excluded (n=68). In addition, patients with a diagnosis of SLE were excluded (n=8) (**Figure 1**). One patient had Schwachman-Diamond syndrome but also had AIC. A total of 154 patients were included in the study and divided among two groups: AIC-PID (n=17, 11%) and AIC-only (n=137, 89%) (**Figure 1**).

### AIC-Only and AIC-PID Groups: Demographics and Diagnosis

The mean age of AIC onset was comparable between the AIC-PID and AIC-only groups (6.9 vs 6.6 years, *p*=0.85) (**Table 1**). There was no significant difference in sex distribution between the two groups (*p*=0.179). Among AIC-PID patients, the PID diagnoses included partial DiGeorge syndrome (pDGS) (n=7), genetically uncharacterized CVID/CID (n=6), genetically defined CID as follows: CHH-RMRP (cartilage-hair hypoplasia secondary to defect in the RNA component of mitochondrial RNA processing endoribonuclease) (n=1), CTLA-4 (cytotoxic T-lymphocyte-associated protein 4 haploinsufficiency (n=1), IKAROS (*IKZF1* gene defect) (n=1), and Kabuki syndrome (*KM2TD* gene defect) (n=1) (**Table 1**). PID was confirmed by genetic testing in 4 of 10 CVID/CID patients (40%). pDGS was confirmed by FISH (fluorescence *in situ* hybridization), either at our institution or by referring physician. In total, 11 out of the 17 patients in the AIC-PID group (65%) were diagnosed with either pDGS or genetically-defined PID. The remaining six patients in the AIC-PID group underwent genetic testing and were found to have no genetic mutations consistent with PID or variants of unknown significance that could completely explain their phenotype. After the study period closed, expanded PID genetic panels became more widely available, and two of these six patients were later diagnosed with Kabuki syndrome, while a third was diagnosed with *CTLA-4* haploinsufficiency.

**Table 1:** Demographic data and diagnosis of AIC-PID and AIC-only patients.

	AIC-PID, n = 17		AIC-only, n = 137	*p*-value
**Sex**	**(n, %)**		**(n, %)**	** **
Male (% of Cohort)	11 (64.7)		65 (47.4)	0.179
Female (% of Cohort)	6 (35.3)		72 (52.6)	
**Age Initial Diagnosis**	**Average (SD)**		**Average (SD)**	
Mean age Dx AIC (years)	6.9 (5.6)		6.6 (5.4)	0.85
** pDGS sub-analysis age at diagnosis**	**w/o pDGS Avg (SD) n = 10**	**pDGS Avg (SD) n = 7**	** **	** **
Mean age Dx AIC (years)	6.7 (5.4)	6.1 (4.8)	**NA**	0.788
Mean age Dx PID (years)	10.2 (6.3)	0.7 (1.5)		0.001*
Difference between PID and AIC Dx (years)	3.5 (4.2)	−5.4 (5.4)		0.006*
**PID Diagnosis**	**n (%)**		** **	** **
Partial DiGeorge syndrome (pDGS)	7 (41.2)		**NA**	
CVID/CID (Not genetically characterized)	6 (35.3)			
IKAROS (*IZKF1*)	1 (5.9)			
Cartilage hair hypoplasia (CHH-*RMRP*)	1(5.9)			
CTLA-4 haploinsufficiency	1 (5.9)			
Kabuki syndrome (*KMT2D*)	1 (5.9)			
**Autoimmune Cytopenia Diagnosis**	**n (%)**		**n (%)**	
AIHA	11 (64.7)		16 (11.7)	<0.0001*
ITP	15 (88.2)		122 (89.1)	0.919
AIN	6 (35.3)		1 (0.7)	<0.0001*
Single-lineage cytopenia	6 (35.3)		134 (97.8)	<0.0001*
Multi-lineage cytopenia	11 (64.7)		3 (2.2)	<0.0001*

AIC, Autoimmune Cytopenia; PID, Primary Immune Deficiency; pDGS, partial DiGeorge Syndrome; CVID/CID, Common Variable Immunodeficiency/Combined Immunodeficiency; CID with G, Combined Immunodeficiency with Genetic Characterization; CHH(RMRP), Cartilage-hair hypoplasia (RNA component of mitochondrial RNA processing endoribonuclease); CTLA-4, Cytotoxic T-lymphocyte-associated protein 4 haploinsufficiency; AIHA, Autoimmune Hemolytic Anemia; ITP, Immune Thrombocytopenia; AIN, Autoimmune Neutropenia; Dx, diagnosis; SD, standard deviation.

*indicates statistically significant.

AIC-PID patients had a higher frequency of AIHA and AIN compared to AIC-only patients (*p*<0.0001, *p*<0.0001 respectively). AIC-PID patients were also more likely to develop Evans syndrome; conversely, AIC-only patients had a higher frequency of single-lineage cytopenias (*p*<0.0001) (**Table 1**).

Since AIC can be a presenting symptom of PID and may precede its diagnosis, we anticipated a delay between the onset of cytopenias and the diagnosis of PID. Among patients in the AIC-PID group, 59% of patients were diagnosed with AIC before the diagnosis of PID (n=10). The remaining 41% were diagnosed with PID before AIC (n=7).

The seven patients who were diagnosed with PID prior to AIC diagnosis had a diagnosis of pDGS. Therefore, we did a subgroup analysis of patients with and without pDGS within the AIC-PID group to compare the timing of initial PID diagnosis to the diagnosis of AIC (**Table 1**). The mean age of PID diagnosis and the difference between PID and AIC time of diagnosis were significantly different between patients with and without pDGS (*p*= 0.001, 0.006 respectively) (**Table 1**). PID was diagnosed 3.5 years *later* than AIC in the non-pDGS group, whereas pDGS patients were known to have PID on average, 5.4 years *before* diagnosis of AIC (*p*=0.006).

### Clinical Presentation

Three clinical features were more frequently observed in the AIC-PID group as compared to the AIC only group (**Table 2**): splenomegaly (OR 13.0; 95% CI [3.8-44.4]; *p*=<0.0001), short stature (OR 56.6; 95% CI [6.1-525]; *p*=0.006]), and history of recurrent or chronic infections (OR 23.0; 95% CI [6.9-76.6]; *p*=<0.0001). Lymphadenopathy was also more common in the AIC-PID group and approached statistical significance (OR 3.9; 95% CI [0.92-17.1]; *p*=0.06). In contrast, bleeding/bruising/petechiae was more common in the AIC-only group (OR 0.36; 95% CI [0.13-1.0]; *p*=0.05). There was no significant difference between the history of fever or jaundice in the two groups (**Table 2**). No summary measures were calculated for failure to thrive due to zero events in the AIC-only group.

**Table 2:** Clinical presentation of AIC-PID and AIC-only patients.

Clinical presentation	AIC-PID, n = 17 (n, %)	AIC-only, n = 137 (n %)	Univariate OR, [95% CI], *P-*value	Adjusted OR, [95% CI], *P*-value
Splenomegaly	7 (41.2)	7 (5.1)	13.0, [3.8-44.4], <0.0001*	12.4, [2.8-54.2], 0.001*
Short stature	5 (29.4)	1 (0.7)	56.6, [6.1-525.2], 0.006*	58.2, [5.3-634], 0.001*
History of failure to thrive	2 (11.8)	0 (0.0)	**	**
History of recurrent/chronic infections	10 (58.8)	8 (5.8)	23.0, [6.9-76.6], <0.0001*	24.1, [5.65-103.4], <0.0001*
Lymphadenopathy	3 (17.6)	7 (5.1)	3.9, [0.92-17.1], 0.064	7.9, [1.68-37.6], 0.009*
Bleeding/bruising/petechiae	9 (52.9)	104 (75.9)	0.36, [0.13-1.0], 0.05*	0.32, [0.086-1.164], 0.084
Fever	1 (5.9)	15 (10.9)	0.53, [0.06-4.1],0.508	0.90, [0.11-7.6], 0.926
Jaundice	1 (5.9)	13 (9.5)	0.60, [0.07-4.86], 0.596	1.06, [0.124-9.04], 0.958

*statistically significant; **no summary measures were calculated due to zero events in some cells.

Univariate OR applies to logistic regression performed on full data set (AIC-PID vs. AIC-only). Adjusted OR adjusts for pDGS status.

*indicates statistically significant.

Because patients with partial DiGeorge syndrome may have unique features including short stature, a logistic regression analysis was done comparing AIC-only to AIC-PID groups, controlling for pDGS. The results remain largely unchanged when adjusted for pDGS status. Controlling for pDGS, splenomegaly, short stature, and history of recurrent or chronic infections remained features that distinguished the AIC-PID group. Adjusted odds ratios can be found in **Table 2**. Lymphadenopathy (LAD) distinguished AIC-PID from AIC-only when controlling for pDGS (OR 7.9; 95% CI [1.68-37.6]; *p*= 0.009). Bleeding/bruising/petechiae no longer distinguished AIC-only from AIC-PID patients when controlling for pDGS diagnosis.

### Immune Phenotyping

The proportion of patients with at least one immune panel tested (cellular or humoral immune evaluation) was higher in the AIC-PID group than in the AIC-only group (100% vs 12.4%; p<0.0001). Comparison of lymphocyte subsets between the groups is summarized in **Table 3**. Low CD3 and CD8 counts distinguished the AIC-PID group from the AIC-only group (p=0.005 and p=0.04 respectively) (**Table 3**). Lymphocyte proliferation studies to mitogens and antigens were not obtained in the AIC-only group; therefore, they were not compared between groups.

**Table 3:** Immunology testing in AIC-PID and AIC-only patients.

Immune Panels	AIC-PID (n = 17), n (%)	AIC-only (n = 137), n (%)	*P-*value
Immune Tested (one or more panels)	17 (100)	17 (12.4)	<0.0001*
**Cellular Immune Evaluation**	**AIC-PID, n (%)**	**AIC-only, n (%)**	
**Lymphocyte Subsets**	**(tested n = 16)**	**(tested n = 15)**	
Low CD3 (T cell)	10 (62.5)	2 (13.3)	0.005*
Low CD4 (T cell)	7 (43.8)	4 (26.7)	0.32
Low CD8 (T cell)	6 (37.5)	1 (6.7)	0.040*
Low CD19 (B cell)	5 (31.3)	1 (6.7)	0.083
Low CD56 (NK cell)	6 (37.5)	3 (20.0)	0.283
**Lymphocyte Proliferation to Mitogen**	**(tested n = 11)**	**(tested n = 0)**	
Low PHA	3 (27)		
Low Con-A	5 (45)		
Low PWM	2 (18)		
**Lymphocyte Proliferation to Antigen**	**(tested n = 9)**	**(tested n = 0)**	
Low Candida	5 (55)		
Low Tetanus	1 (11)		
**Humoral Immune Evaluation**	**(tested n = 16)**	**(tested n = 17)**	
Low IgG	10 (62.5)	1 (5.9)	0.001*
	**(tested n = 16)**	**(tested n = 16)**	
Low IgA	10 (62.5)	4 (25)	0.033*
Low IgM	8 (50.0)	7 (43.8)	0.723
**Vaccine Titers**	**(tested n = 7)**	**(tested n = 1)**	
Low Tetanus Titer	2 (28)	0 (0)	
	**(tested n = 8)**	**(tested n = 1)**	
Low Diphtheria Titer	4 (50)	0 (0)	
	**(tested n = 9)**	**(tested n = 1)**	
Low Pneumococcal Titer	7 (78)	1 (100)	

Lymphocyte subsets (CD3, CD4, CD8, CD19, and CD56), lymphocyte proliferation studies to mitogens and antigens, immunoglobulins (IgG, IgA, IgM), and vaccine titers were recorded. Pre- vs. post-vaccine pneumococcal titers are not documented as dates of vaccination were not searchable in the electronic medical record. Test results were recorded from patients for whom they were available.

CD, Cluster of differentiation; NK, Natural Killer Cells; PHA, phytohemagglutinin; Con A, concanavalin; PWM, pokeweed mitogen. *Indicates statistically significant results.

Within the AIC-PID group, 62.5% (n=10) patients had low initial IgG values (low for age and not drawn while on immune globulin replacement) compared to 5.9% (n=1) of the AIC-only group. IgA values (adjusted for age) were also lower in the AIC-PID group, with 62.5% (n=10) of the AIC-PID group having low IgA values compared to 25% (n=4) of those tested for IgA in the AIC-only group (*p*=0.033). Only one patient in the AIC-only group had vaccine titers documented, which prohibited a comparison between the two groups. Expert guidelines recommend that titers be obtained prior to initiation of immunoglobulin replacement therapy when clinically feasible (22). In cases of severe or life-threatening cytopenias, it may not be clinically feasible to perform diagnostic studies of antibody specificity, as IVIG may be initiated on an emergency basis, preempting vaccine titer testing. In addition, AIC diagnosis preceded PID diagnosis by several years in many cases.

Autoantibody profiling is summarized in **Table 4**. Serologic testing for any autoantibody to blood cells (Coombs test, anti-platelet antibodies and/or anti-neutrophil antibodies) was more commonly performed in the AIC-PID group compared to AIC-only group (n=16/17, 94.1% vs. n=81/137 59.1%, *p*=0.005). The AIC-PID group had a higher fraction of patients with autoantibodies to red blood cells and to platelets among those tested for each type of autoantibody (*p*=0.0002, p=0.036, respectively). AIC-PID patients were more likely to have two or more auto-antibodies present compared to AIC-only patients (*p*=0.0002). A comparison of the prevalence of anti-neutrophil antibodies between the two groups could not be made as only 2-3 patients in each group were tested.

**Table 4:** Autoantibody testing to blood cells of AIC-PID and AIC-only groups.

Antibody Tests	AIC-PID (%)	AIC-only (%)	*p*-value
Any autoantibody tested/total cohort	16/17 (94.1)	81/137 (59.1)	0.005*
Presence of any auto-antibodies/among those tested	13/16 (81.3)	40/81 (49.3)	0.019*
Positive Coombs/tested for Coombs	11/12 (91.7)	20/58 (34.4)	0.0002*
Presence of/tested for anti-platelet antibody	6/8 (75)	21/38 (55.2)	0.036*
Presence of/tested for anti-neutrophil antibody	3/3 (100)	2/3 (66.7)	n/a
Presence of two or more antibodies	5/17 (29.4)	3/81 (3.7)	0.0002*

*Indicates statistically significant results.

### Treatments and Outcomes

**Figure 2:**
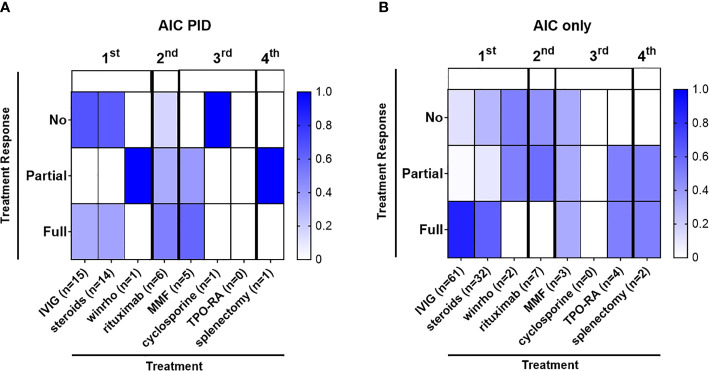
Autoimmune cytopenias in the AIC-PID group are often refractory to first line therapy. The diagram depicts the distribution of patients with varying degrees of response to treatment (percentage of patients with no, partial or full response to first, second, third and fourth line treatment shown by color gradient as indicated); therapeutic grouping by first line (IVIG, corticosteroids, Rho(D) immune globulin), second-line (rituximab), third line (mycophenolate mofetil [MMF], cyclosporine), adjunct thrombopoietin receptor agonists [TPO-RA]) and fourth-line treatment (splenectomy) for the AIC-PID and AIC-only groups. **(A)** Patients treated in the AIC-PID group, (n = 15) 88.2% of AIC-PID patients. **(B)** Patients treated in the AIC-only group, (n = 76) 55% of AIC-only patients.

Some patients with AIC-PID did not require treatment due to the transient and/or mild nature of their AIC. In the AIC-PID group, only 5.8% of patients (1/17) did not require treatment, versus 45% (61/137) in the AIC only group. Patients with AIC-PID were more likely to receive first line therapy versus no therapy compared to patients in the AIC-only group (88.2% vs 55%, *p*=0.01 respectively). Among patients treated with first line therapy, a larger fraction of patients failed first line therapy within the AIC-PID group (n=10/15, 66%) compared to the AIC-only group (n=14/76, 18%, *p*<0.0001) (**Table 5**). Fifty-three percent (53%) of the AIC-PID group (n=9/17) received second- or third-line therapy compared to only 8% of the AIC-only patients (n=11, *p*<0.0001). Rituximab was used as second line therapy in a greater percentage of patients in the AIC-PID group versus the AIC-only group (41.2% [n=7/17] vs 5.8% (n=8/137]) (**Table 5**). Among patients in the AIC-PID group, 60% had a full response, and 40% had a partial response to rituximab (**Figure 2**). Patients in the AIC-only group tended not to respond as well to rituximab (40% no response; 60% partial response). T-cell directed therapy with MMF led to a partial or full response to therapy in 5 AIC-PID patients (**Figure 2**), while the three AIC-only patients who received MMF had a mixed response (1 no response, 1 partial, and 1 full response). In the AIC-PID group, 1 patient underwent splenectomy without a lasting response; two patients in the AIC-only group underwent splenectomy with partial or full response. No patients in the AIC-PID group received adjunct thrombopoietin receptor agonists (TPO-RA) agents, eltrombopag or romiplostim. In the AIC-only group, 4 patients received eltrombopag, with partial or full response.

**Table 5:** Treatment response in the AIC-PID and AIC-only groups.

Treatment	AIC-PID, n = 17 (n, %)	AIC-only, n = 137 (n, %)	*p*-value
No treatment	1 (5.8)	61 (45.0)	0.01*
Received 1st-Line Treatment	15 (88.2)	76 (55.0)	0.01*
Treated with Steroids	15 (88.2)	33 (24.1)	<0.0001*
Failed 1st Line Treatment	10 (58.8)	14 (10.2)	<0.0001*
Received 2nd or 3rd Line Treatment	9 (52.9)	11 (8.0)	<0.0001*
Treated with Rituximab	7 (41.2)	8 (5.8)	<0.0001*
Splenectomy	1 (5.8)	2 (0.01)	n/a
Adjunct agents (eltrombopag/romiplostim)	none	4 (0.03)	n/a

First-line therapies (IVIG, corticosteroids, Rho(D) immune globulin or combination), second-line (rituximab) and third-line (mycophenolate mofetil [MMF], cyclosporine); adjunct agents thrombopoietin receptor agonists eltrombopag and romiplostim [TPO-RA]. *symbol indicates statistical significance.

Comparison of ITP patients in the AIC-PID versus AIC-only group for treatment refractoriness revealed that AIC-PID patients with ITP were more treatment refractory than those in the AIC-only group. In the AIC-PID group, there were 15 patients with ITP; 14/15 were treated with first line therapy (93%). Of these, 10/14 (71%) failed first line therapy. By contrast, in the AIC-only group, there were 122 patients with ITP; among these, 77 were treated with first line therapy (63%). Of these, only 10 failed first line therapy (13%). Therefore, a much larger percentage of patients in the AIC-PID group required first line therapy for ITP (93% vs 63% in the AIC-only group), and a larger percentage of AIC-PID ITP patients failed first line therapy (71% vs 13%).

## Discussion

In this retrospective study, clinical features that help to distinguish AIC-PID patients from patients with AIC only include splenomegaly, short stature, and history of recurrent or chronic infections. Bleeding/bruising/petechiae was more common in the AIC-only group, which may reflect the prominence of acute ITP in this group. Controlling for partial DiGeorge syndrome, lymphadenopathy also distinguished between AIC-PID and AIC-only groups.

Our study suggests that lymphocyte subsets and immunoglobulin testing may help identify patients with underlying PID who deserve further immune evaluation including genetic testing. AIC-PID patients had lower T-cell counts (CD3 and CD8 T cells) and lower immunoglobulin levels (IgG and IgA) compared to AIC-only patients. Therefore, immunoglobulin levels and lymphocyte subsets could be helpful for identifying patients who warrant further evaluation for underlying PID.

While this study suggests that at least 11% of patients presenting with AIC may have underlying PID, treatment-refractoriness may offer some additional guidance regarding which patients require additional screening for PID. AIC-PID patients were more likely to fail first line therapy than AIC-only patients (58.8% vs 10.2%). Among patients with ITP, which is less strongly associated with PID than AIHA or Evans syndrome, a larger percentage of ITP patients in the AIC-PID group required first line therapy (93% vs 63% in the AIC-only group). AIC-PID ITP patients were also more likely to fail first line therapy (71% vs 13%). For clinicians, this suggests that screening laboratory tests would be higher yield for diagnosing PID in ITP patients who are refractory. PID screening could therefore be reserved for ITP patients who have persistent or refractory ITP. AIHA, AIN, and Evans syndrome are more strongly associated with PID. PID screening might be beneficial in patients with at least one episode of AIHA, AIN, or Evans Syndrome.

Retrospective chart review is inherently limited. Limitations of the study include the fact that during this study, PID genetic testing panels were limited to a few genes, and understanding of AIC as a presenting sign of PID was not widely appreciated. Further, genetic testing was performed on only a subset of patients due to the nature of the study; for example, patients treated by hematologists who did not refer to immunology did not have access to PID genetic testing. The proportion of patients with AIC-PID is likely underestimated as a result. Referrals to immunology specialists at a tertiary care center during the time period of this study may have been more skewed toward patients with congenital anomalies such as pDGS and Kabuki syndrome, because syndromic physical features facilitate diagnosis in the absence of genetic testing. This may partially explain the proportion of pDGS patients in this cohort. Fortunately, genetic testing for PIDs has expanded, and AIC is now more widely understood to be a presenting sign of PID (23). One positive outcome of the study at our center was to raise awareness among non-immunologists that AIC can be a presenting sign of PID. A forthcoming prospective AIC-PID study will demonstrate increased collaboration between immunology and hematology and broader genetic testing of the prospectively enrolled subjects.

Partial DiGeorge patients with cytopenias, especially severe refractory cytopenias, should not be overlooked. A large pDGS cohort (67 patients) is followed at our center. Of these 67 pDGS patients, 26/67 (39%) have any type of cytopenia. Eight of the 67 (11%) have AIC; two have severe refractory cytopenias requiring hospitalization and long-term immune suppression. While severe autoimmune cytopenias in pDGS have been reported (24–27), and a recent study of 130 pDGS patients (28) noted nearly 4% of pDGS patients (5/130) to have AIC, the proclivity to severe refractory AIC in some pDGS patients may not be widely appreciated. This study corroborates findings that pDGS patients can develop severe refractory autoimmune cytopenias.

Because subjects, laboratory evaluations, and treatments were captured by chart review, authors had no jurisdiction regarding the clinical management or testing of the subjects that were not directly under their care. Immune evaluation was partial or not pursued for most of the patients in the AIC-only group. Patients managed by non-immunologists tend not to undergo full immune phenotyping. This could be because an underlying immune disorder was not suspected, as the link between AIC and PID was not widely appreciated, or features suggestive of underlying PID were not present.

Eleven patients in the AIC-PID group had genetically confirmed PID. DiGeorge syndrome (*del22q11*) was most common, followed by mutations in *CTLA4, RMRP, IKZF1, and KMT2D*. These diagnoses were made in 2013-2016 before large genetic panels for PID were widely available. Other studies of Evans syndrome in PID also support the utility of genetic testing for PID in AIC patients (10, 29–31). Evans syndrome has been associated with genes that cause autoimmune lymphoproliferative syndrome ALPS, (32), mutations in signal transduction and activator of transcription 3 (*STAT3*) (33), tripeptidyl peptidase 2 (*TPP2*) (34) and other immune genetic defects. A 2018 review by Rotz et al. summarizes genetic defects linked to chronic refractory AIC including *CTLA-4*, nuclear factor kappa B subunit 1 (*NFKB1*)*, STAT3*, and several genes that cause ALPS, among others (29). Genetic testing for underlying PID can guide decision-making regarding the best available therapy for PID patients.

SLE has long been associated with monogenic complement defects (35). Interestingly, several additional genes that cause PID have recently been linked to monogenic SLE and other autoimmune/autoinflammatory disorders (1, 35–40). We might not have excluded SLE patients if we had begun this study today. Polygenic autoimmune and autoinflammatory disorders may be attributed to a combination of immune genetic defects as well (40).

It is possible that among the AIC-only group, there were patients with undetected PID, as not all patients received full immune evaluation, because they were not all referred to immunology. The small sample size of AIC-PID patients also limits conclusions that can be drawn from this study. A forthcoming prospective AIC cohort study from our group is likely to further distill the characteristics that distinguish AIC-PID patients from AIC-only patients.

Hematologists and immunologists should pursue investigation of PID among AIC patients with signs of immune dysregulation. Collaboration between hematologists, rheumatologists, and immunologists to distinguish subpopulations of patients with AIC by their immune phenotype, autoantibodies, and clinical features will likely improve treatment regimens and clinical outcomes. Early identification of AIC patients with PID may reduce treatment failure, morbidity, mortality, and costs of treatment. Prospective studies are needed to develop a comprehensive evaluation and treatment strategy targeting the immunopathology of AIC.

## Data Availability Statement

The raw data supporting the conclusions of this article will be made available by the authors, without undue reservation.

## Ethics Statement

The studies involving human participants were reviewed and approved by Johns Hopkins All Children’s Hospital Institutional Review Board. Written informed consent from the participants’ legal guardian/next of kin was not required to participate in this study in accordance with national legislation and the institutional requirements.

## Author Contributions

EW-C assumed authorship of study in 2018 for purpose of revising manuscript, re-analyzing data, and coordinating submission. CM collected the majority of data *via* chart review, and participated in data analysis and writing of manuscript. AM participated in chart review and initiated writing of manuscript. JD provided critical feedback and review of manuscript, suggested additional analyses. DA was involved in early statistical analysis. ME participated in study coordination and review of manuscript. BP participated in chart review and writing. MB, CH, JMa, JMe, JL, PS, and IA assisted in study development, management of AIC patients, and review of manuscript. AK performed statistical analysis. JW conceived the study, secured funding, participated in manuscript revision and review of data, and supervised the work. All authors contributed to the article and approved the submitted version.

## Funding

This research was partly funded by Johns Hopkins All Children’s Hospital (JHACH) Institutional Grant entitled “Feasibility study to assess the role of T and B cells in refractory cytopenias in children” (JW), the Jeffrey Modell Foundation, Jeffrey Modell Diagnostic and Research Center at JHACH, and the Robert A. Good Endowment at the University of South Florida.

## Conflict of Interest

The authors declare that the research was conducted in the absence of any commercial or financial relationships that could be construed as a potential conflict of interest.

## Abbreviations

ALPS, autoimmune lymphoproliferative syndrome; AIC, autoimmune cytopenia; AIHA, autoimmune hemolytic anemia; AIN, autoimmune neutropenia; CHH, cartilage hair hypoplasia; CID, combined immunodeficiency; CTLA-4, cytotoxic T-lymphocyte antigen 4; CVID, common variable immunodeficiency; ES, Evans syndrome; IgG/IgA/IgM, immunoglobulin G, A, or M; IKZF1, IKAROS Family Zinc Finger 1; KMT2D, Lysine methyltransferase 2D; ITP, immune thrombocytopenic purpura; MMF, mycophenolate mofetil; MRN, medical record number; PID, primary immunodeficiency; pDGS, partial DiGeorge syndrome; RMRP, RNA component of mitochondrial RNA processing endoribonuclease; SLE, systemic lupus erythematosus; TCR, T-cell receptor; TPO-RA, thrombopoietin receptor agonist; STAT3, signal transduction and activator of transcription 3; TPP2, tripeptidyl peptidase 2; NFKB1, nuclear factor kappa B subunit 1.
